# Research Literature on the Intersection of Dementia, Spirituality, and Palliative Care: A Scoping Review

**DOI:** 10.1093/geroni/igaa057.3187

**Published:** 2020-12-16

**Authors:** Jennifer Palmer, Alyssa Smith, Sara Paasche-Orlow, George Fitchett

**Affiliations:** 1 Hebrew SeniorLife, Boston, Massachusetts, United States; 2 Independently Employed, Bethesda, Maryland, United States; 3 Hebrew SeniorLife, Roslindale, Massachusetts, United States; 4 Rush University Medical Center, Chicago, Illinois, United States

## Abstract

Dementia marks an increasingly prevalent terminal illness for which palliative care, including spiritual care, could improve quality of life. Research gaps exist in understanding the intersection of dementia, spirituality, and palliative care. Thus, we conducted the first scoping review examining the nature and breadth of peer-reviewed studies across these three topics. The scoping review followed methods from The Joanna Briggs Institute Reviewers’ Manual (2015). We developed a priori a scoping review protocol outlining the Population, Concept and Context for study, data sources, search strategy, inclusion/exclusion criteria, and procedure for screening, extracting, and analyzing data. The final sample consisted of 19 studies with the following themes: Characterizing Spiritual Needs, Preferences, and Resources; Characterizing Palliative or Spiritual Care; Predicting Provision of Spiritual Care; and Assessing Spiritual Care Interventions. Eighteen studies were published in the past decade, and eleven were based in Europe. The majority of studies focused on long-term care settings, grouped stages of dementia or did not specify dementia stage, and investigated interventions indirectly related to spiritual palliation. Many studies were limited in sample size and in generalizability / transferability and used less sophisticated research designs. Accordingly, research across dementia, spirituality, and palliative care needs to examine distinct stages of dementia; hospital-, home- and community-based settings; and formal spiritual care interventions (e.g., administered by chaplains) and needs to utilize rigorous study designs (e.g., randomized clinical trials). Such research could advance practice and policy that enhance quality of life for tens of millions of persons with dementia and their family members worldwide.

